# Automating provision of feedback to stroke patients with and without information on compensatory movements: A pilot study

**DOI:** 10.3389/fnhum.2022.918804

**Published:** 2022-08-08

**Authors:** Daphne Fruchter, Ronit Feingold Polak, Sigal Berman, Shelly Levy-Tzedek

**Affiliations:** ^1^Department of Industrial Engineering and Management, Ben-Gurion University of the Negev, Beer-Sheva, Israel; ^2^Recanati School for Community Health Professions, Department of Physical Therapy, Faculty of Health Sciences, Ben-Gurion University of the Negev, Beer-Sheva, Israel; ^3^Herzog Medical Center, Jerusalem, Israel; ^4^Zlotowski Center for Neuroscience, Ben-Gurion University, Beer-Sheva, Israel; ^5^Freiburg Institute for Advanced Studies (FRIAS), University of Freiburg, Freiburg, Germany

**Keywords:** stroke, exergames, user experience, patient-centered design, compensatory movements, human-computer interface, serious games, human-machine interface

## Abstract

Providing effective feedback to patients in a rehabilitation training program is essential. As technologies are being developed to support patient training, they need to be able to provide the users with feedback on their performance. As there are various aspects on which feedback can be given (e.g., task success and presence of compensatory movements), it is important to ensure that users are not overwhelmed by too much information given too frequently by the assistive technology. We created a rule-based set of guidelines for the desired hierarchy, timing, and content of feedback to be used when stroke patients train with an upper-limb exercise platform which we developed. The feedback applies to both success on task completion and to the execution of compensatory movements, and is based on input collected from clinicians in a previous study. We recruited 11 stroke patients 1–72 months from injury onset. Ten participants completed the training; each trained with the rehabilitation platform in two configurations: with motor feedback (MF) and with no motor feedback (control condition) (CT). The two conditions were identical, except for the feedback content provided: in both conditions they received feedback on task success; in the MF condition they also received feedback on making undesired compensatory movements during the task. Participants preferred the configuration that provided feedback on both task success and quality of movement (MF). This pilot experiment demonstrates the feasibility of a system providing both task-success and movement-quality feedback to patients based on a decision tree which we developed.

## Introduction

### Movement compensations performed by individuals post-stroke

Individuals who have had a stroke and suffer from sensorimotor impairments in the upper limb may use compensatory movement patterns in their everyday lives (Cirstea and Levin, 2000; Liebermann et al., 2012).

For example, a person who cannot extend their elbow to reach an object located within their arm’s reach may bend their trunk to get to that object. Repeatedly performing such compensatory movements may play a central role in impairing recovery following a stroke (Cirstea and Levin, 2000; Shaikh et al., 2014). That is because once an undesirable movement pattern is learned, it is hard to unlearn that pattern and replace it with one that is more desirable (Alaverdashvili et al., 2008), a phenomenon that has been called “learned bad use” (Alaverdashvili and Whishaw, 2013). Since individuals may not always be aware of the compensatory movements they make, individuals post-stroke need to receive feedback on whether they are performing undesired compensatory movements whenever they engage in rehabilitation exercises, and receive guidance on how to accomplish a motor task without performing undesirable movements (Levin and Demers, 2021). While clinicians provide such feedback to their patients in an individual one-on-one session, the gap between the demand for specialized clinicians (e.g., physical therapists and occupational therapists) and the available resources (e.g., availability of clinicians for one-on-one sessions) continues to widen (Hancock, 2007); Technological tools are thus being developed to help narrow this gap [e.g., (Veerbeek et al., 2017)], for example, by providing instructions and feedback to patients during their training process (Feingold-Polak et al., 2021a). The goal of such technological tools is to enable patients to practice their rehabilitation exercises at home or in the clinic, even when a clinician is not available for a one-on-one therapy session. It is therefore paramount that such technological platforms would be able to provide feedback to patients on their compensatory movements. This, in turn, should be done without overwhelming the patients with too much information, which could lead to the patients’ frustration (Fruchter et al., 2022). We are not aware of any currently available schemes for an automated decision-making process, to enable assistive technology to methodically provide relevant information on compensatory movements.

We developed a rehabilitation platform which provides individuals post-stroke with feedback on task success (Feingold-Polak et al., 2021a). Our ultimate goal is that this platform would also provide feedback to patients on the way they perform their movement, using an automated compensation-detection algorithm we developed (Kashi et al., 2020); It will thus benefit both self-training and in-clinic training by: (1) providing feedback to patients on their movement patterns, so that they can correct their movements during practice, with the potential to increase their range of motion and reduce pain over the long term (Sigrist et al., 2013); (2) tracking the presence of compensatory movements over time, and reporting it to the clinician; this will provide an unbiased assessment of the patient’s progress when practicing on their own without the presence of the clinician, allowing for better adaptation of the individual in-clinic sessions to the patient’s needs and progress.

As a first step toward achieving this goal, we aimed to test in the current study whether it is feasible to provide patients with complex feedback, combining information on task success and on movement compensations, without overwhelming the patients with information. To that end, we used the Wizard-of-Oz approach (Riek, 2012; Rea et al., 2017): a clinician identified the movement compensations, and inputted them into the platform; the platform then chose the appropriate feedback to provide to the patients, based on a decision tree we developed.

Providing feedback on various aspects of the training process (task success, the presence of several compensatory movements) is a nuanced task that therapists navigate based on their training and experience. We thus first conducted, in a previous study, a set of four focus-group discussions with a total of 20 stroke clinicians, to understand their approach to providing feedback to patients within a practice session (Fruchter et al., 2022).

### Feedback

It has been suggested that meaningful feedback should be provided during or after practicing rehabilitation exercises, both on the outcome of the movement and on its quality (Levin and Demers, 2021). Feedback can be given on different aspects of the movement: its speed, smoothness, precision, variability, and coordination, to name a few (Levin et al., 2009; Levin and Demers, 2021). It can be simple – e.g., different sounds to indicate success/failure on a task – or detailed – e.g., a verbal or visual explanation of the error and how to correct it (Van Vliet and Wulf, 2006; Schmidt and Wrisberg, 2008). Feedback can be classified as either “intrinsic” [available *via* one’s internal sensory system, through vision, proprioception, touch pressure, and auditory perception (Levin and Demers, 2021)], or “extrinsic” (usually provided by an external source; e.g., a therapist indicating the quality of a person’s movement). Extrinsic feedback has been categorized into either “knowledge of results” (KR) or “knowledge of performance” (KP) (Van Vliet and Wulf, 2006). Knowledge of results provides information on task completion (Magill and Anderson, 2010); For example, lifting an object and placing it in the indicated location. KP provides information on the movement parameters (Magill and Anderson, 2010); For example, when patients flex their trunk when reaching for an object, a therapist may show them how to correctly perform the movement, and advise them, to maintain contact between their trunk and the back of the chair, to avoid trunk flexion. It has been suggested that KP is more effective than KR in terms of motor-learning outcomes and retention of learned movement patterns for post-stroke individuals in the chronic stage (Subramanian et al., 2010). We previously suggested that there are four main parameters to consider when providing feedback: (1) hierarchy; (2) modality; (3) timing; and (4) content (Fruchter et al., 2022). Hierarchy refers to prioritizing information “urgency”: when feedback is given on several aspects of the task’s performance (e.g., both on speed and on the presence of compensatory movements), it is important to determine which is of higher priority, so that users are not overwhelmed by too much information. Modality refers to the sensory modality through which feedback is provided: visual (e.g., a picture on a computer screen indicates the error the user has performed, and how it should be corrected), auditory (e.g., recorded speech explains what should be changed in the movement), or tactile (e.g., a vibrating belt can be used to indicate success/failure on the task) (Lin et al., 2019). Timing refers to when the feedback is given, in relation to the performance: either concurrently (during performance) or terminally (after performance) (Schmidt and Wrisberg, 2008; Magill and Anderson, 2010). There is no consensus on the optimal timing of feedback in the context of stroke rehabilitation (Molier et al., 2010).

Content refers to the type of information provided (e.g., on movement speed vs. on compensations).

A recent study (Lin et al., 2019) examined the feasibility and acceptability of visual feedback on reducing compensatory movements during stroke rehabilitation exercises. Participants saw a video mirroring their movements, showing either direct footage from a video camera recording the patients’ movements, or an avatar representation of the patients’ body (rendered as a collection of rectangular blocks). Most participants stated that the system functioned as a mirror does, in the absence of active detection of compensatory movements, and noted that a system that would automatically detect compensations and alert them when a compensation was performed, using auditory and/or visual cues would be helpful (Lin et al., 2019).

### Individualized feedback

An important principle in rehabilitation is that practice should be progressive and optimally tailored to the individual’s capabilities (Van Vliet and Wulf, 2006; Kleim and Jones, 2008; Langhorne et al., 2009; Winstein and Kay, 2015). In conventional post-stroke rehabilitation, the therapist provides verbal and visual feedback, and typically uses hands-on demonstration (Hartveld and Hegarty, 1996; Ballinger et al., 1999; DeJong et al., 2004; Wohlin Wottrich et al., 2004). However, when using technological systems that are unable to adapt and change feedback modality, timing, and content to meet the needs of each individual, it is difficult to provide feedback that is individually tailored to each patient (Parker et al., 2011).

As a first step in developing an individually tailored rehabilitation platform, we conducted here a pilot study with individuals post-stroke. They exercised with a rehabilitation platform we developed (Feingold-Polak et al., 2021a), and received feedback based on an adaptive decision tree. The decision tree implemented a feedback hierarchy which we inferred from focus groups we held with clinicians in a previous study (Fruchter et al., 2022). Participants received feedback on both task success and on the quality of their movement. In a cross-over design, in the control condition they received feedback only on task success. Our goal in the current study was to test the feasibility and applicability of providing feedback both on task success and on movement quality (presence or absence of compensatory movements) to individuals post stroke.

The main contributions of this paper are: (1) The implementation of a hierarchical feedback scheme – which is based on therapists’ clinical decision process – on a rehabilitation platform for individuals post stroke; (2) collection and analysis of input from individuals post-stroke on using this platform – with and without feedback on compensatory movements they performed.

## Materials and methods

### Participants

A total of 11 individuals who have had a stroke (five women, six men; age range 45–77 years, mean 63.4 ± 10.6; 1–72 months from injury onset, mean 14.6 ± 21.5; FMA score 30–54/60, mean 45.4 ± 7.7) participated in this study. Participants who met the following inclusion criteria were recruited to the study: (1) Diagnosis of acquired brain injury (ABI), unilateral stroke (ischemic or hemorrhagic), traumatic brain injury, or brain tumors as confirmed by imaging data from hospital discharge records; (2) Age 18–85 years; (3) Mini-Mental State Examination (MMSE) score ≥ 24/30 (for participants ≥ 65 years) (Folstein et al., 1975) or the equivalent Montreal Cognitive Assessment (MoCA) score ≥ 23/30 (for participants < 65 years) (Carson et al., 2018); (4) Fugl-Meyer Upper Extremity (FM-UE) score 16–54/60 [higher scores indicated less impairment of the paretic upper limb; a score below 16/60 indicates the patient does not have the capacity to reach and grasp objects, and a score above 54/60 means the impairment is too mild for the exercise platform to be relevant (Feingold-Polak et al., 2021a)]; (5) No excessive pain in the affected upper limb, defined as ≤4 on a 10-point Visual Analog Scale (VAS). Exclusion criteria for the study group were as follows: (1) Additional neurological or musculoskeletal problems (such as Parkinson’s disease, unilateral neglect, Pusher syndrome and apraxia); (2) Severe vision deficits which would limit the ability to view the computer screen, or sensory deficits affecting upper limb movements; (3) Aphasia impeding comprehension of simple instructions (Levin et al., 2016). The participants were recruited from the Adi Negev rehabilitation center, from the ambulatory day care and from the department with the help of the clinical team, supervised by an MD specializing in Physical Medicine and Rehabilitation. All participants gave their written informed consent to take part in this study. The protocol was approved by the Tel HaShomer Helsinki committee.

### Procedure

Each participant took part in one 35–55-min session, which consisted of two experimental conditions, in a single-subject AB/BA crossover design (Josephy et al., 2015). Each condition consisted of 12 trials and lasted between 10 and 25 min, depending on the participant’s ability and fatigue. The two experimental conditions were: with motor feedback (MF), and with no motor feedback (control condition) (CT). The two conditions were identical, except for the feedback content provided: in both conditions they received feedback on task success; in the MF condition they also received feedback on making compensatory movements during the task. The order in which the conditions were presented was semi-randomized, such that half of the participants started with the MF condition, and half with the CT condition.

#### Basic protocol

**FIGURE 1:**
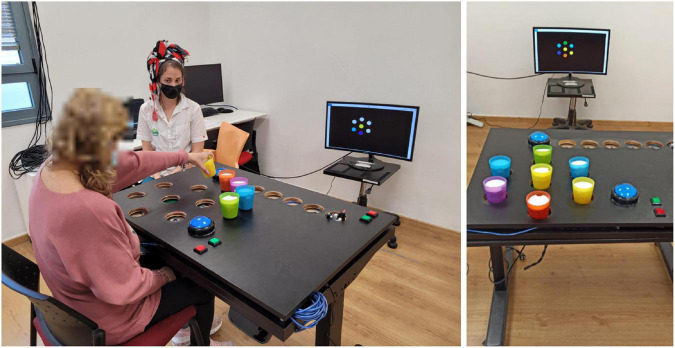
The experimental setup. *Left:* The participant was seated in front of a height-adjustable table, behind which was a 27-inch computer screen, on which instructions and feedback were presented. The clinician sat in the same room, next to a second computer, used to monitor the experimental progression and input information about movement compensations that the participant performed. In each of 12 trials, a set of colored circles was displayed on the computer screen, arranged in a circle around a central location, similar to a bullseye arrangement. The participant had to place a corresponding set of colored cups on the table according to the picture shown on the computer’s screen. *Right:* The Target Exercise Game with all seven cup locations occupied, arranged according to the on-screen instructions.

**FIGURE 2:**
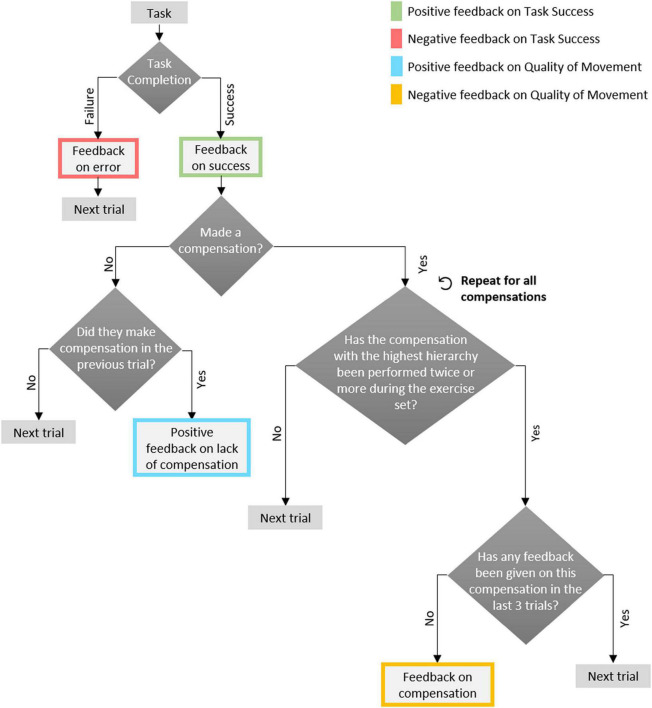
The decision tree used in the experiment. It is depicted as a flow chart, and is based on the hierarchical structure suggested by clinicians in a previous study (Fruchter et al., 2022): feedback on task success precedes feedback on compensations; within compensations, order is set by the clinician (here, set to a default order: trunk flexion, scapular elevation and elbow flexion).

In both conditions the participants performed a functional exercise, designed to practice reach-grasp-and-place movements of their upper limb. Specifically, they sat in an armless chair in front of a height-adjustable table, behind which was a 27-inch computer screen, approximately 2.15m from the participant (see Figure 1). The clinician sat in the same room, next to a second computer, used to monitor the experimental progression and input information about movement compensations that the participant performed (see details below). In each trial, a set of colored circles was displayed on the computer screen; they were arranged in a ring, with up to six colored circles arranged around a single circle in the middle of the ring (see Figure 1). The participant had to place a corresponding set of colored cups on the table according to the picture shown on the computer’s screen (Feingold-Polak et al., 2021a). There were four levels of game difficulty, depending on the number of cups, starting from three cups in the first (easiest) level, up to seven cups in the fourth (most challenging) level; the cups weighed 180g each. The height-adjustable table was fit with a custom-built top plate, with holes 8-cm in diameter, so that the patients could comfortably place the cups in the designated locations, without the risk of knocking them over. This exercise set requires spatial perception and a multidimensional movement of the hand in different directions across the plane of the table (sideways, and forward-back), and calls for upper-limb functional reach-to-grasp (RTG) movements. This exercise set corresponds to the “Target Game” described in Feingold-Polak et al. (2021a). In both the MT and the CT conditions, feedback on task success was provided in each trial (e.g., “well done! You are playing great!” or “You arranged it wrong this time; no worries, let’s continue”). In the MF condition, feedback on movement quality was also provided, based on a decision tree, as detailed below (see Figure 2).

#### Sensing apparatus

The exercise platform detected where the cups were placed on the table by using an Arduino Mega board, with RFID readers. The RFID readers, placed below the 8-cm holes in the top platform, identified the tags that were attached to the cups (on the inside). The participant pressed a large push button (9cm in diameter) to indicate they completed the task, at which point the data were processed by the Arduino board and transported to the computer via a USB cable. They received feedback from the computer within 3–4 s from pushing the response button (Feingold-Polak et al., 2021a).

Compensation detection The detection of the compensatory movements was done by a professional clinician, an occupational therapist who treats individuals with acquired brain injury in her daily practice, using the Wizard-of-Oz approach (Riek, 2012; Rea et al., 2017). Thus, the participants had the impression that the system detected their movements autonomously, not knowing that the clinician was the one who detected the compensatory movements. Using a custom-built graphical-user interface (GUI), the clinician marked which of the following three compensations were performed by the participant in each trial: trunk flexion, scapular elevation, and elbow flexion. The compensatory movements performed by the participants were recorded in both conditions; feedback on them was provided to the participants only in the MF condition.

#### Motor feedback

In the MF condition, in addition to feedback on task success, participants received real-time feedback on the quality of their movement; That is, on whether they performed compensatory movements, and how they should modify their movement to avoid compensation. We endeavored to provide the feedback – in terms of content and frequency – such that it is helpful to the individuals, and does not overburden them. To that end, in a previous study, we conducted four focus groups with 20 rehabilitation clinicians, and based on their input we created a rule-based set of guidelines for the desired hierarchy, timing, content and modality of feedback such the training system should provide (Fruchter et al., 2022). Here, we used this rule set to build a decision tree (Figure 2) for provision of feedback to participants; it is based on the following principles: (1) feedback on task success is most important, and is always provided (for every trial); (2) only if the participant completed the task correctly, information may be given on compensatory movements for that trial; (3) information is given at most on a single compensation per trial, even if multiple compensations were performed; (4) the hierarchy of compensations (which to give feedback on before the others) was set to the following default order: trunk flexion, scapular elevation and elbow flexion; (5) feedback on a compensation is given only after it was performed on at least two trials during the exercise set; (6) After a trial for which feedback on a compensation was given, no compensation-related feedback is given on that particular compensation, during the following three trials, so as to give patients the opportunity to correct their movements without relying on external feedback; (7) if the participant completed the task successfully, and without performing any compensation, after they did perform one in the previous trial (whether or not they received feedback on it in the previous trial), they receive positive feedback on avoiding the compensatory movement. Feedback was thus provided on successful (e.g., “you succeeded!”) or unsuccessful (”you were not right, but try again!”) completion of the task, and on the presence of a compensatory movement (e.g., “When you extend your hand forward, please straighten your elbow and avoid bending your trunk”) or its absence (e.g., “Well done! Your trunk remained straight as you extended your hand forward”).

#### Custom-built questionnaires

**FIGURE 3:**
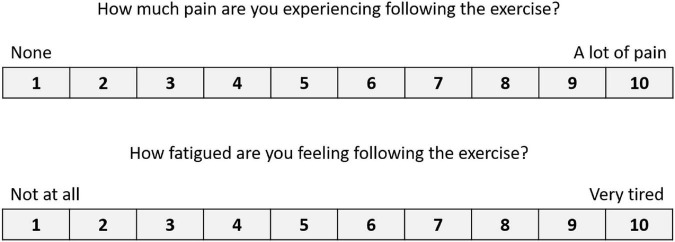
Numeric scales to indicate levels of pain and fatigue. Used following both the CT and the MF conditions.

After each condition, the participants answered an 11-item (CT) or an 18-item (MF) usability questionnaire, based on the System Usability Scale (SUS) (Brooke, 2013) (see Tables 1, 2, respectively); they indicated their responses using a five-point scale from (1) “Strongly disagree” to (5) “Strongly Agree.” The items included reverse-wording to improve validity and control for acquiescence (Weijters and Baumgartner, 2012). Since some of the participants had cognitive impairments due to their injury, the clinician who ran the experiment guided all participants through the questionnaires, to ensure that they understood all the questions. In addition, we asked the participants open-ended questions to allow them to describe their experience with the system in detail (see Table 3). After answering all the questions, the participants were asked to rate their pain level on a scale from (1) “None” to (10) “A lot of pain,” and their fatigue level on a scale from (1) “Not at all” to (10) “very tired” (see Figure 3).

**TABLE 1:** Questions presented at the end of the control condition (CT) [based on the system usability scale (SUS) questionnaire].

1. I would like to use this system frequently
2. The feedback the system provided was clear
3. I think that I would need assistance to be able to use this system
4. I would imagine that most people would learn to use this system quickly
5. I think exercising with the system over time can help my rehabilitation process
6. I felt very confident using the system
7. I was overall satisfied with the use of the system
8. I think the system provides too much feedback on task success
9. I think the feedback the system provided on task performance is sufficient
10. I do not think the feedback the system provides will help me complete the task better

**TABLE 2:** Questions presented at the end of the motor feedback (MF) condition [based on the system usability scale (SUS) questionnaire].

1. I would like to use this system frequently
2. The feedback the system provided was clear
3. I knew how to change my body movement according to the feedback I received from the system (for example, when the system said “Pay attention, you are bending your trunk”)
4. I think that I would need assistance to be able to use this system
5. I would imagine that most people would learn to use this system quickly
6. I think the system successfully tracked my body movements
7. I think the feedback the system provides will also help me perform tasks outside the treatment room
8. I think exercising with the system over time can help my rehabilitation process
9. I felt very confident using the system
10. I was overall satisfied with the use of the system
11. In my opinion there was a balance between the feedback given on the quality of movement and the feedback given on task success
12. I think the system gave too much feedback on task success
13. I think the feedback the system provided on task success is sufficient
14. I do not think the feedback the system provides will help me complete the task better
15. I think the system provided too much feedback on the quality of movement
16. I think the feedback the system provided on quality of movement is sufficient
17. I did not understand the feedback that was given on the quality of movement
18. I do not think the feedback the system provides will help me perform better-quality movements

**TABLE 3:** Custom-made open-ended questionnaire.

1. What do you think are the advantages of the system?
2. What do you think are the disadvantages of the system?
3. Did you find anything missing in the system? (Would you have liked to add anything to it?)
4. To what extent, in your opinion, the feedback on movement quality contributes to the exercise session?
5. What did you think of the feedback the system provided?
6. Which exercise set did you prefer? The one where you received feedback on task success only (e.g., “well done, you ordered the cups correctly”), or the one where you received feedback on task success and on your movements (e.g., “pay attention, you raised your shoulder”).
7. Do you have any further comments?

### Data analysis

Data were analyzed using SPSS (Statistical Packages for Social Sciences, 26.0). We used the Wilcoxon Signed-rank test to analyze and compare the number of compensatory movements that were made in each condition (CT and MF), and to analyze the different domains of the custom-build usability questionnaires across the two conditions (CT and MF), significance levels were set at p < 0.05. Reverse worded items were reversed coded to (5) “Strongly disagree” and (1) “Strongly agree” before analysis (Matlock et al., 2018). Participants’ open-ended responses were analyzed as a proportion of respondents holding a certain perspective. We used the Wilcoxon Signed-rank test to analyze the different levels of pain and fatigue across the two conditions, and we used repeated-measures ANOVA to check if there’s a difference between pain and fatigue levels across the order of the conditions.

## Results

Table 4 presents the demographic characteristics of the participants. All the participants in the study had had a stroke; they were either at the subacute or the chronic stage (Kim and Winstein, 2017). Three participants had their non-dominant side affected; they used their affected (non-dominant) arm to complete the task. Of the 11 participants in this study, one woman dropped out from the study after completing the first part of the experiment (the MF condition; participant 2 in Table 4). She was fatigued, and did not wish to continue the exercise set. We report here the analysis of the data from the 10 participants who completed the study. Timing and compensation records for one participant were lost due to a technical error. One participant (participant 9 in Table 4) completed the two exercise sets in two separate sessions, as she complained of high pain levels after one session (the MF condition). She reported pain levels of 10 on the VAS scale after both sessions.

**TABLE 4:** Demographic characteristics of the participants.

Participant	Age	Gender	Months since injury	Rehabilitation framework	MMSE	Fugl-Meyer	Dominant hand	Side of the body affected by stroke
1	55	Woman	3	In-patient	21/30	40/60	L	L
2 – dropped out	74	Woman	2	In-patient	24/30	R:41/60 L: 58/60	R	R&L
3	46	Man	10	Out-patient	27/30	43/60	R	R
4	66	Man	11	Out-patient	23/30	52/60	R	R
5	67	Man	1	Out-patient	23/30	46/60	R	R
6	68	Woman	6	Out-patient	23/30	54/66	R	L
7	77	Man	3	Out-patient	28/30	38/60	L	R
8	45	Man	3	None	23/30	30/60	R	R
9	72	Woman	13	Out-patient	21/30	52/60	R	L
10	64	Woman	72	Out-patient	21/30	49/60	R	R
11	63	Man	36	Out-patient	21/30	54/60	R	R

### Timing and number of compensations performed in the control condition and in the motor feedback condition

It took participants an average of 15.7 ± 7.5 min to complete the exercise set in the CT condition, and 15.1 ± 4.3 min in the MF condition.

The total number of compensations performed in the CT condition (mean = 13.0 ± 15.6) was not significantly different (Wilcoxon Signed-Rank test, *Z* = −1.057, *p* = 0.291) from the total number of compensations performed in the MF condition (mean = 11.9 ± 12.7). On the individual compensatory-movement level, the compensation rates of *trunk flexion* (CT: 6.1 ± 5.6; MF: 5.3 ± 6.0; Z = −1.289, *p* = 0.197) and *elbow flexion* (CT: 3.3 ± 5.9; MF: 1.6 ± 2.8; Z = −1.461, *p* = 0.144) were higher in the CT condition, while the compensation rate of *scapular elevation* (CT: 3.6 ± 5.4; MF: 5.0 ± 5.3 in the MF, *Z* = −1.511, *p* = 0.131) was higher in the MF condition.

### The modified system usability scale questionnaire

**FIGURE 4:**
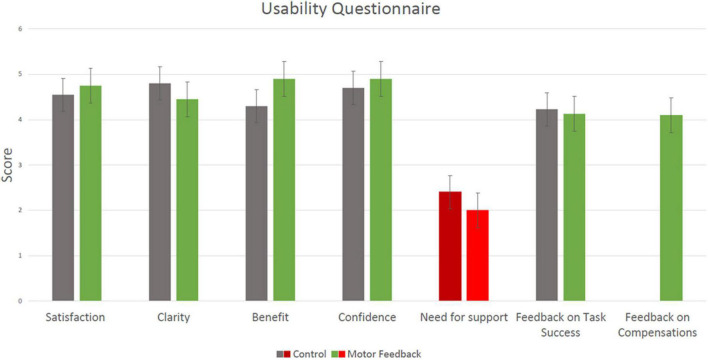
Results of the 11-item modified System Usability Scale (SUS) questionnaire (mean ± SD). A score of 1 reflects “Strongly disagree” and 5 reflects “Strongly agree.” Results from the CT condition are marked in gray and results from the Motor-Feedback condition are marked in green; The “need for support” category is marked with a different color, as it was negatively worded: a lower score corresponds to higher usability of the platform. The rightmost category (“Feedback on compensations”) shows a single bar, since feedback on compensations was provided only in the MF condition.

Results of the 11-item modified SUS questionnaire are summarized in Figure 4. We grouped the statements into 6 categories: Satisfaction, Clarity, Benefit, Confidence, Need for support and Feedback. The mean score for the *satisfaction* category, which consisted of the statements “I would like to use this system frequently” and “I was overall satisfied with the use of the system” was 4.6 ± 1.0 out of 5 for the CT condition and 4.8 ± 0.9 for the MF condition (*Z* = −1.633, *p* = 0.102). The mean score for the *clarity* category, which consisted of the statements “The feedback the system provided was clear” and” I would imagine that most people would learn to use this system quickly” was 4.8 ± 0.5 out of 5 for the CT condition and 4.5 ± 0.8 for the MF condition (*Z* = −1.588, *p* = 0.112). The mean score for the *benefit* category, which consisted of the statement “I think exercising with the system over time can help my rehabilitation process” was 4.3 ± 1.3 out of 5 for the CT condition and 4.9 ± 0.3 for the MF condition (Z = −1.289, *p* = 0.197). The mean score for the *confidence* category, which consisted of the statements “I felt very confident using the system” was 4.7 ± 0.7 out of 5 for the CT condition and 4.9 ± 0.3 for the MF condition (*Z* = −0.816, *p* = 0.414). The mean score for the *need for support* category, which consisted of the statements “I think that I would need assistance to be able to use this system” was 2.4 ± 1.8 out of 5 for the CT condition and 2.0 ± 1.7 for the MF condition (*Z* = −1.633, *p* = 0.102). Within the *feedback* category, the mean score for the statements “I think the system provided too much feedback on task success,” “I think the feedback the system provided on task success is sufficient” and “I do not think the feedback the system provides will help me complete the task better” was 4.2 ± 1.1 out of 5 for the CT condition and 4.1 ± 1.4 for the MF condition (*Z* = −0.321, *p* = 0.748). In the MF condition, the *feedback* category also included the statements “I think the system provided too much feedback on the quality of movement,” “I think the feedback the system provided on quality of movement is sufficient” and “I do not think the feedback the system provides will help me perform better-quality movements,” and the mean score for those was 4.1 ± 1.3 out of 5. The results thus did not show a significant difference between the two conditions in all six categories, and indicate an overall high level of satisfaction from the platform in both conditions.

**FIGURE 5:**
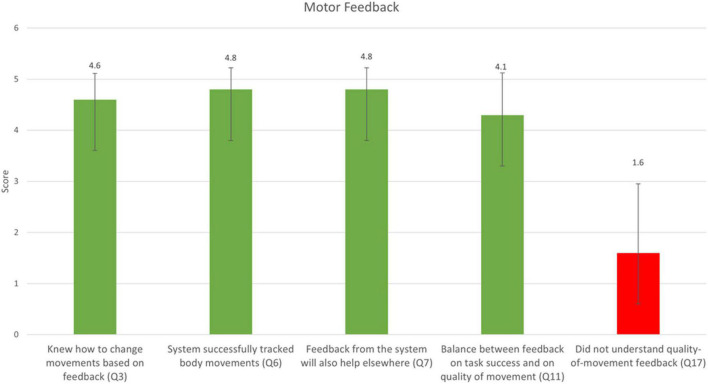
Responses to the five statements that were only presented in the MF condition (mean ± SD). A score of 1 reflects “Strongly disagree” and 5 reflects “Strongly agree.” The question numbers correspond to the questions listed in Table 2. The rightmost bar is marked with a different color, as it was negatively worded: a lower score corresponds to higher usability of the platform.

Responses to the five statements that were presented to the participants following the MF condition only are shown in Figure 5. The mean score for the statement, “I knew how to change my body movement according to the feedback I received from the system (for example, when the system said ‘Pay attention, you are bending your trunk’)?” was 4.6 ± 0.5 out of 5. The mean score for the question, “I think the system successfully tracked my body movements” was 4.8 ± 0.4 out of 5. The mean score for the statement, “I think the feedback from the system will also help me perform tasks outside the treatment room” was 4.8 ± 0.4 out of 5. The mean score for the statement “In my opinion, there was a balance between the feedback given on the quality of movement and the feedback given on task success” was 4.3 ± 0.8 out of 5. The mean score for the statement “I did not understand the feedback that was given on the quality of movement” was 1.6 ± 1.3 out of 5. The results thus indicate that participants found the instructions in the MF condition over movement quality to be overall clear and balanced with respect to feedback on task success.

### Results from the pain and fatigue level scales

The self-reported levels of fatigue (CT: 4.2 ± 3.0; MF: 5.8 ± 2.7; *Z* = −0.841, *p* = 0.4) and pain (CT: 3.9 ± 3.2; MF: 5.6 ± 3.1; *Z* = −1.36, *p* = 0.174) were lower in the CT condition, though a Wilcoxon signed-rank test indicated that these differences were not significant.

We performed a repeated-measures ANOVA to test whether the reported fatigue levels at the end of a condition were affected by the relative timing of this condition [that is, whether the condition was performed first (S1) or second (S2)]. Indeed, there was a statistically significant difference in the reported fatigue levels between S1 and S2 [*F*(1,33) = 7.27, *p* < 0.031], such that higher fatigue levels were reported following S2.

Similarly, we performed a repeated-measures ANOVA to test whether the reported pain levels at the end of a condition were affected by the relative timing of this condition. Here, too, we found a statistically significant difference in reported pain levels between S1 and S2 [*F*(1,21) = 9.82, *p* < 0.016], such that higher pain levels were reported following S2.

### Results from the open-ended questionnaire

Most (9 out of 10) participants stated that they prefer to receive feedback on both task success and movement quality, as opposed to feedback on task success only (question #6 in the open-ended questionnaire; see Table 3). Seven participants noted that the feedback on movement quality was very helpful and contributed to the exercise (question #5), and six said that, in their opinion, nothing is missing in the system (question #3). One participant (P8) noted that the feedback on quality of movement had a little contribution to the exercise. He noted that he has an engineering background, and that he felt the buttons on the table are too close to the body, making them easy to press by mistake. He further suggested displaying a timer on the screen, as well as proactively offering breaks after a few trials. He, as well as participant P6, noted that if feedback is given, it should be compatible with their abilities; specifically, they *must* lift their shoulder in order to reach forward with their hand, so they could not avoid making this compensation (implying that feedback on scapular elevation was not useful to them).

Participants noted several benefits of the system (question #1): Three participants said that the advantage of the system is that it combines physical work of the hand with cognitive work. Four participants said that the system supports intensive hand work, four participants said that the system activates the mind, and three participants said that the system provides clear instructions. One participant said she liked that the system gave instructions on quality of movement.

*“Both the brain and the hand work” (P1)*

*“By using the system, I can practice a lot with my hand” (P3)*

*“[the system allows for] speed of thought, precise execution of the task and color detection” (P7)*

*“After it [the system] told me I needed to straighten my back, I actually did it” (P9)*

One participant (P7) noted that practice with the system was too short (he noted it at the end of the first condition, which was MF in this case). This participant also noted there was a technical problem with the system: the participant placed the cups correctly, but the system gave incorrect feedback. Three participants (P7, P9, and P10) suggested making the task more challenging by adding additional object colors and using different objects, other than cups. One participant (P11) noted that feedback on quality of movement is missing in the system after exercising in the CT condition (the MF condition was first for him).

## Discussion

We implemented a feedback algorithm based on input from clinicians onto a gamified rehabilitation-exercise platform, and conducted a pilot study with individuals who have had a stroke. We demonstrated the feasibility and acceptability of the proposed feedback structure for providing individualized real-time feedback to stroke patients training with the platform. Participants gave high ratings to both configurations of the platform (providing feedback only on task success vs. providing feedback on both task success and compensatory movements); Nine out of 10 preferred the configuration that provided feedback on both task success *and* on quality of movement.

In the current study we used the Wizard-of-Oz approach, whereby an occupational therapist identified and recorded the compensatory movements performed by the participants; In the future, this process of compensation detection can be automated using a machine-learning algorithm we developed for automatic identification of compensatory movements (Kashi et al., 2020).

### An assessment of the system’s usability with and without feedback on movement quality

The participants’ usability assessment of the system was similar in both conditions. Specifically, participants’ satisfaction levels were high and similar in both conditions (CT and MF), which support the fact that adding movement quality feedback does not impair the motivation to practice with the system. Indeed, in their responses to the open-ended questionnaire, nine out of 10 participants preferred the condition that included motor feedback.

Participants also appreciated the combination of motor and cognitive elements in the exercise set. Three participants noted it as an advantage of the system, and three participants expressed their desire to experience more challenging exercises. These results are in line with research showing that game-based exercises that are competitive or cooperative increase patient learning, motivation, confidence, and positivity (Matarić et al., 2007; Hatem et al., 2016; Feingold-Polak et al., 2021a).

### Patients prefer to get a combination of task-success and movement-quality feedback

We found that most the participants (nine out of 10) preferred to receive feedback on both task success and movement quality. This indicates that the feedback structure we used, based on the decision tree shown in Figure 2 was not overwhelming for them, and indeed, was perceived as helpful. In line with these results, previous studies found that patients indicated that a system that would automatically detect compensations, notify them when compensations are performed, and provide feedback according to their motor and cognitive performance would be helpful (Lin et al., 2019; Feingold-Polak et al., 2021a).

### Evaluation of the feedback on quality of movement

It was previously found that providing too much information can detract learners from focusing on the most relevant information (Eaves et al., 2011). To avoid this, we previously held focus groups with clinicians (Fruchter et al., 2022), to learn about the structure and the content of the feedback they provide during a session. Based on their input, we built a decision tree (Figure 2), which we implemented in the current experiment. The participants’ overall impression from the MF condition was positive, and they indicated that there was a balance between the feedback on task success and the feedback on the quality of the movement. This suggests that the intervals we built into providing feedback, based on the decision tree, were appropriate, in terms of user perception.

As the clinicians in our previous study (Fruchter et al., 2022) noted, a therapist who knows the patient’s capabilities should determine in advance what compensations the system will provide feedback on, such that the feedback is tailored to each user individually. Indeed, one of the participants in the current study received feedback on compensatory movement even though he could not perform the task without this movement; this led to his dissatisfaction with receiving irrelevant feedback. This outcome underscores the need to tailor the feedback content based on the individual’s capabilities.

### Effect of motor feedback on compensatory movements: Future directions

In this pilot experiment, our goal was to test the feasibility of implementing the decision tree for provision of feedback, based on clinicians’ input. As such, we did not expect to find a change in the number of compensations performed within a single exercise session. The non-significant difference between the CT and the MF conditions could be affected by the small number of participants in the pilot study and by the fact that each participated in a single session per condition (CT/MF). Yet another underlying cause is suggested in a comment made by two of the participants, noting that the feedback provided on shoulder movement (i.e., scapular elevation) was not useful to them, since they must perform this compensation to complete the exercise. Thus, the participants could not avoid this compensation and it may explain the high occurrence rate of this compensation. The effect of providing movement-quality feedback on the number of compensations performed should be further explored in a multi-session study, with a larger number of participants.

It has been demonstrated that muscle fatigue can increase the likelihood of compensatory behavior (Cirstea and Levin, 2000). In the current study results show that all participants rated their fatigue and pain levels higher on the second condition, regardless of which one it was (CT/MF). This may have affected the compensation rate in both conditions. It is thus advisable to hold shorter, single-condition exercise sessions in future experiments, and allow for breaks within a session, as needed (Feingold-Polak et al., 2021a).

One of the study participants was color-blind, and he had difficulty distinguishing between the blue and purple cups, and the red and green cups. In a future study we recommend marking the cups with an additional element other than color (e.g., shape/printed text on them), to allow color-blind participants to benefit from this exercise platform.

In its current configuration, the platform provides feedback only during the session; it will be helpful – both to patients and to their clinicians – to have this information available over time: that is, to be able to track the user’s performance over many sessions (e.g., in terms of compensations performed, time to complete a session, etc.) using a simple graphical user interface (GUI). We are currently building this functionality into the platform.

Finally, it is advisable to include a short video (to be displayed on the computer screen) demonstrating the movement that needs to be corrected, the first time the system provides feedback on quality of movement (for each compensation type). Thus, patients with more pronounced cognitive impairments, such as those with aphasia, will be able to benefit from the system’s quality-of-movement feedback.

While the goal of the current work was to test the feasibility of a platform that informs patients of their compensatory movements, along with information on their success on the task, this platform may provide as additional benefit when used over time: it is conceivable that providing patients with feedback on compensatory movements even when they train in the absence of a clinician, may hone their ability to detect their own compensatory movements during practice, and perhaps even outside the practice sessions. This will have to be tested empirically in future work.

In the Introduction section, we noted the widening gap between the need for specialized clinicians and the available resources, and the development of new technological tools to help narrow this gap. It is our goal that a platform such as the one presented here – which provides patients with instructions for their exercise, and layered feedback on their performance – will be a stepping stone in the process of bridging this gap.

### Study limitations

As this was a pilot study to test the feasibility and usability of the platform, the sample size was small, participants were heterogeneous in terms of the time that had passed since the stroke, and they underwent a single session in each of the conditions (with/without feedback on movement-quality). A study with more participants and more sessions per participant will be necessary to gain a more thorough understanding of the benefit of motor feedback provided by such a rehabilitation platform, and to establish the applicability of the findings over the long term; Division of users into the subacute and the chronic stages will enable asserting the effects of the intervention at the different stages, as well as their potentially different satisfaction levels from the platform. In the current study, the detection of compensatory movements was performed by a clinician who was familiar with the compensatory patterns of five of the participants. Future studies aiming to establish the change in the patterns of compensatory movements over time would benefit from using an automatic detection of compensatory movements (Kashi et al., 2020). Three of the participants in the current study used their non-dominant (affected) arm to complete the task. It has been documented that this affects the performance of the task [e.g., the mean force exerted is higher (Feingold-Polak et al., 2021b)]. It remains to be examined in future work whether using the non-dominant side also affects parameters such as satisfaction from the system.

## Conclusion

We developed and tested a scheme for automating the decision-making process for provision of feedback to stroke patients by assistive technology; Specifically, we tested patient preferences when the rehabilitation platform we developed provided feedback only on task success vs. combined feedback on task success and on movement compensations. Patients gave high scores to both configurations on the usability questionnaire, and nine out of 10 indicated their preference to receive the combined feedback on task success and on movement compensations. This system, when coupled with automated compensation-detection capabilities, will enable individuals post-stroke to receive complex feedback when training in between sessions with the clinician (e.g., during in-home exercise).

## Data availability statement

The raw data supporting the conclusions of this article will be made available by the authors, without undue reservation.

## Ethics statement

The studies involving human participants were reviewed and approved by The Chaim Sheba Medical Center (Tel HaShomer). The patients/participants provided their written informed consent to participate in this study. Written informed consent was obtained from the individual(s) for the publication of any potentially identifiable images or data included in this article.

## Author contributions

DF, RF, SB, and SL-T contributed to conception and design of the study. DF orchestrated the execution of the study, analyzed the data, and wrote the first draft of the manuscript. SL-T supervised the project, secured the funding for it, and wrote the final version of the manuscript. All authors contributed to manuscript revision, read, and approved the submitted version.
